# Intestinal microbial dysbiosis aggravates the progression of Alzheimer’s disease in *Drosophila*

**DOI:** 10.1038/s41467-017-00040-6

**Published:** 2017-06-20

**Authors:** Shih-Cheng Wu, Zih-Syuan Cao, Kuo-Ming Chang, Jyh-Lyh Juang

**Affiliations:** 1 Institute of Molecular and Genomic Medicine, National Health Research Institutes, Zhunan, Miaoli 35053 Taiwan; 2 Department of Pathology, Hsinchu Mackay Memorial Hospital, Hsinchu, 30071 Taiwan; 3 Ph.D. Program for Aging, China Medical University, Taichung, 40402 Taiwan

## Abstract

Neuroinflammation caused by local deposits of Aβ_42_ in the brain is key for the pathogenesis and progression of Alzheimer’s disease. However, inflammation in the brain is not always a response to local primary insults. Gut microbiota dysbiosis, which is recently emerging as a risk factor for psychiatric disorders, can also initiate a brain inflammatory response. It still remains unclear however, whether enteric dysbiosis also contributes to Alzheimer’s disease. Here we show that in a *Drosophila* Alzheimer’s disease model, enterobacteria infection exacerbated progression of Alzheimer’s disease by promoting immune hemocyte recruitment to the brain, thereby provoking TNF-JNK mediated neurodegeneration. Genetic depletion of hemocytes attenuates neuroinflammation and alleviated neurodegeneration. We further found that enteric infection increases the motility of the hemocytes, making them more readily attracted to the brain with an elevated oxidative stress status. This work highlights the importance of gut–brain crosstalk as a fundamental regulatory system in modulating Alzheimer’s disease neurodegeneration.

## Abstract (web summary)

Emerging evidence suggests that gut microbiota influences immune function in the brain and may play a role in neurological diseases. Here, the authors offer in vivo evidence from a *Drosophila* model that supports a role for gut microbiota in modulating the progression of Alzheimer’s disease.

## Introduction

Alzheimer’s disease (AD) is a biologically complex neurodegenerative disease with no known single cause. Among various proposed mechanisms for AD pathogenesis, the amyloid cascade is a compelling hypothesis suggesting that pathological accumulation of Aβ_42_ in the brain triggers inflammatory response and oxidative damage in AD^1^. This hypothesis has driven drug development strategies for over 20 years. However, clinical trials of over 100 drugs targeting amyloid build-up in the brain have failed to inhibit the progression of AD, leading the field to consider the use of non-amyloid targeting therapies instead^2, 3^.

There is emerging evidence that gut microbiota dysbiosis is functionally linked to immune dysfunctions of the brain, subsequently contributing to impaired mental health^4^. The intestine has been referred to as the body’s “second brain” because it shares many similar neuronal functions of the brain, affecting mental and emotional well-being^5–7^. The gut and brain are known to functionally affect each other as well. Although it has been hypothesized that dysbiosis of intestinal microbiota can affect the risk or progression of neurological diseases^4^, no in vivo studies have investigated the impact of gut dysbiosis on AD.

Inter-organ communication is an important and conserved mechanism underlying the maintenance of body homeostasis, thus dysregulation of the systemic homeostatic system can give rise to metabolic and neurological disorders^8, 9^. However, studies of the role of inter-organ communication in disease and its underlying signaling mechanisms have only recently just emerged. Studies on model organisms like *Drosophila* have provided valuable insights into the mechanisms essential for our understanding of inter-organ communication during normal or pathological events^10–15^. Therefore, we used a *Drosophila* AD model to investigate the possible involvement of the intestinal infection in neurodegeneration. Comprehensive genetic and phenotypic analysis demonstrated that enterobacteria infection strongly exacerbated neurodegeneration via immune hemocyte recruitment to the brain. In all, this study provides support for the role of interplay between the gut and brain in modulating AD progression.

## Results

### Enteric dysbiosis aggravates AD neurodegeneration in fly

**Fig. 1:**
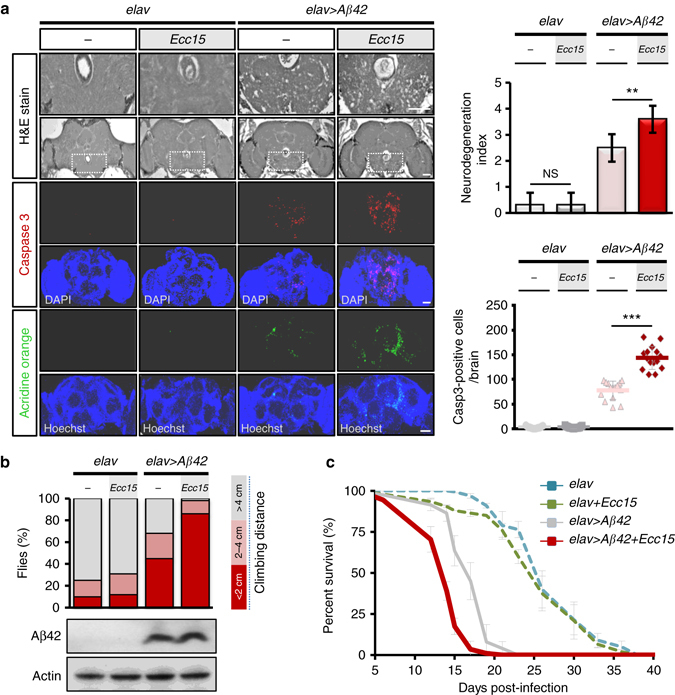
Enteric dysbiosis aggravates neurodegeneration in a *Drosophila* Alzheimer’s disease (AD) model. **a** Histology analysis of degenerated brain tissues (brain section; 14 days postinfection, dpi; *n* = 10) and immunohistochemistry staining of apoptosis markers (whole brain; 10 dpi; *n* = 15). **b**, **c** Negative geotaxis assay of locomotion behavior (*n* = 120 in each group) **b** and lifespan analysis (*n* = 100 in each group) **c**. *elav* >*Aβ42* transgenic or control (*elav* alone) flies intestinally infected with or without *Ecc15*. Error bars represent the SD. The definition of neurodegeneration index is shown in figure legend of Supplementary Fig. 1. H&E, haematoxylin and eosin. **P < 0.01, ***P < 0.001; NS not significant. Scale bars, 50 μm

We induced enteric dysbiosis by oral infection with a non-pathogenic enterobacteria (*Ecc15*), which can persistently colonize the gastrointestinal tract^16^, in adult flies ectopically expressing human Aβ_42_ in their brains^17^. Remarkably, the brains of the infected amyloid transgenic flies displayed a significant increase of vacuolar degeneration compared to controls without infection, while infection did not cause noticeable effects on neurodegeneration in control flies carrying Gal4 alone (Fig. 1a, Supplementary Fig. 1). These results suggested that intestinal infection led to increased neuronal loss in the transgenic brain. This result was further supported by the observed increase in apoptosis as revealed by immunostaining of the brain with apoptosis markers, including active caspase-3, TUNEL and acridine orange in the transgenic flies (Fig. 1a, Supplementary Fig. 2). Life span and locomotor activity are two important measures of AD symptoms, which are known to be significantly reduced in AD. Concomitant to the increased neurodegeneration, locomotor activity and lifespan were also found to be declined in the amyloid transgenic flies subjected to enteric infection (Fig. 1b, c). One concern regarding these results was that the observed neurodegeneration-related phenotypes were actually caused by a direct enterobacteria infection spreading to the brain via a leaky intestinal barrier. We assessed intestinal permeability after enterobacteria infection using a non-absorbable blue food dye, and found no noticeable changes in intestinal permeability (Supplementary Fig. 3). We also found no live *Ecc15* bacteria in the brains after enteric infection (Supplementary Fig. 4), further clarifying that no direct *Ecc15* infection occurred in the brain. To exclude the possibility that these observations might just be an atypical phenomenon of host responses to a particular enterobacteria, we infected the transgenic flies with another enterobacteria *Pseudomonas entomophila* and observed similar phenotypic alterations (Supplementary Fig. 5). These results strongly suggest that the persistent enteric infection is importantly involved in modulating AD neurodegeneration in *Drosophila*.

### Intestinal infection exacerbates inflammation in AD brains

**Fig. 2:**
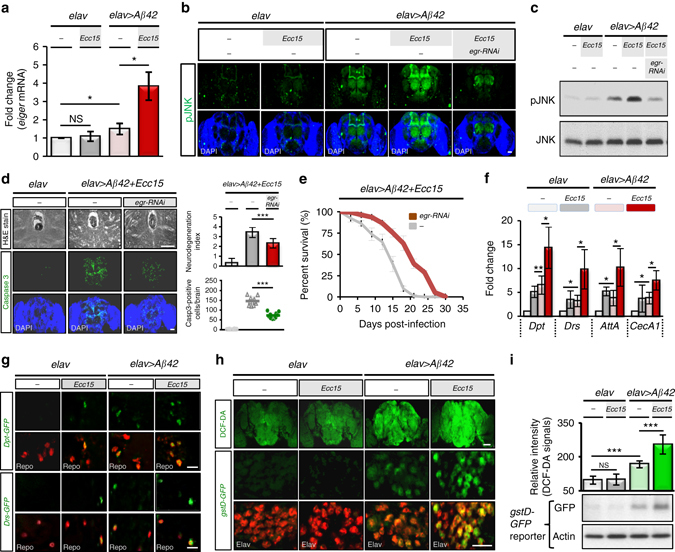
Enteric dysbiosis remotely triggers inflammatory responses in amyloid transgenic fly brain. **a**–**c** *eiger* expression and JNK activity in brains of amyloid transgenic flies with or without *Ecc15* infection. *eiger* mRNA expression analyzed by quantitative RT-PCR (qRT-PCR) **a**, JNK phosphorylation/activation analyzed by immunostaining **b** and western blotting **c** with anti-phospho-JNK antibody. **d**, **e** Neurodegeneration **d** and lifespan **e** analysis after enteric *Ecc15* infection, in Eiger-depleted brain (*elav* > *egr-RNAi*). **f**, **g** qRT-PCR **f** and confocal microscopy **g** analysis of brain AMP levels after *Ecc15* enteric infection. Repo staining (*red*), glial cells; GFP signals (*green*) of reporter gene expressions in *Dpt*- and *Drs-GFP* lines. **h**, **i** Brain oxidative stress after enteric infection. Confocal imaging of DCF-DA (*green*) and Elav (*red*, neuronal cells) signals **h**. Quantification of DCF-DA fluorescent signals (*upper* panel) and western blot analysis of *gstD-GFP* reporter expressions with anti-GFP antibody (lower panel) in **i**. *elav* > *Aβ42* transgenic or control (*elav* alone) flies with or without *Ecc15* intestinal infection assayed at 10 dpi for *eiger* expression, AMP response and ROS stress. Error bars represent the SD of at least three independent experiments. *P < 0.05, **P < 0.01, ***P < 0.001; NS, not significant. H&E, haematoxylin and eosin. *Dpt*, *Diptericin*. *Drs*, *Drosomycin*. *AttA*, *Attacin-A. CecA1*, *Cecropin A1. gstD, glutathione S-transferase D1*. Scale bars, 50 μm **b**, **d** and *upper* **h**; 10 μm **g** and *lower* **h**

Neuroinflammation is widely recognized as a central event in the pathogenesis of AD. Since immune responses in the gut–brain axis are inter-dependent, we investigated whether gut microbial infection would trigger an immune perturbation in the transgenic brain. Among several elevated inflammatory factors, TNF-α is a key proinflammatory cytokine involved in AD development. Early phase clinical trials have suggested that inhibitors against this cytokine may slow down cognitive decline in AD patients^18^. Our results showed that the *Drosophila* TNF *eiger* was readily upregulated in the brain of transgenic Aβ_42_ flies and being subjected to further dysregulation by enteric infection with *Ecc15* (Fig. 2a). Moreover, JNK activity, a known downstream effector of Eiger/TNF signaling which promotes inflammation-induced apoptosis^19, 20^, was also amplified (Fig. 2b, c). To validate that the induction of Eiger is required for JNK activation, we demonstrated that genetic depletion of Eiger from the brain effectively abrogated the activation of JNK and neurodegeneration and prolonged the lifespan of transgenic flies with or without being subjected to enteric infection (Fig. 2b–e, Supplementary Figs. 6 and 7). Because a recent study reported that the over-activation of antimicrobial peptides (AMPs) in the *Drosophila* brain leads to neurodegeneration^21^, we were interested in whether the responses of AMPs would also be affected by intestinal dysbiosis. An analysis of four different AMPs (*Dpt*, *Drs*, *AttA*, and *CecA1*) revealed that all these natural effectors of innate immunity were upregulated in the brains upon enteric infection (Fig. 2f). During AD pathogenesis, glial cells initiate and propagate inflammatory signaling upon the deposition of amyloid in the brain^22^. To determine whether enteric infection also leads to induction of the inflammatory response in glial cells, we combined assays of AMP reporters (*Dpt-GFP* and *Drs-GFP*) and anti-Repo glial cell staining to demonstrate that glial cells were the main triggers of AMP responses in the brain (Fig. 2g). Based on the results that both TNF and AMPs, the two key humoral innate immune components, were highly over-activated in the brain after enteric infection, we conclude that intestinal dysbiosis can remotely stimulate proinflammatory responses in the amyloid transgenic fly brain.

Apart from these humoral inflammatory responses, ROS stress has also been implicated to induce and sustain chronic inflammation in AD. We examined the ROS production in transgenic brain by DCF-DA staining to detect intracellular ROS levels and by *gstD-GFP* reporter assay to quantify ROS activity. Indeed, ROS stress was found to be upregulated after enteric infection (Fig. 2h, i), leading us to conclude that enteric infection induced ROS stress while concomitantly stimulating inflammatory responses in the transgenic brain.

### Enteric infection induces hemocyte recruitment to AD brains

**Fig. 3:**
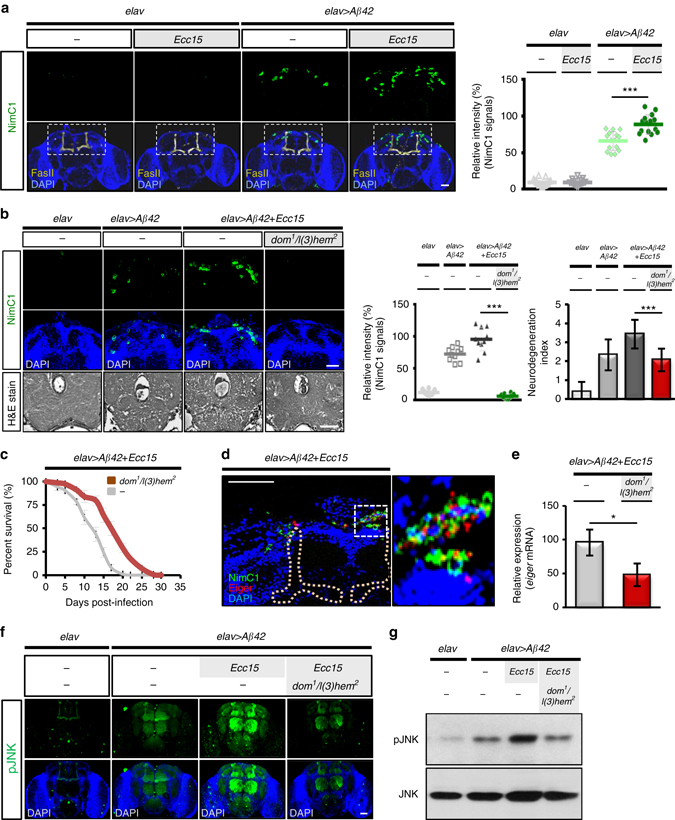
Macrophage recruitment to amyloid transgenic fly brain aggravates neurodegeneration upon intestinal infection. **a** Immunostaining of plasmatocyte (*green*, NimC1 signals) recruited to the transgenic brain upon intestinal infection; *n* = 15 in each group for quantification (*right* panel). *yellow*, mushroom body (FasII signals); *blue*, DAPI signal; *left* panel. **b**, **c** Immunostaining of the recruited hemocytes (*upper* and *middle* panels) and brain histology (*lower* panel) **b** and life span analysis **c** in the transgenic flies with or without *dom* ^1^ */l(3)hem* ^2^ hemocyte-deficient background. *Green*, NimC1 signals; *blue*, DAPI signal; *n* = 10 in each group for quantification of brain histology; *n* = 100 in each group for lifespan assay. **d** Coimmunostaining of NimC1 and Eiger in brains of the *Ecc15*-infected transgenic flies. (*Right* panel shows high magnification view from white dotted line labeled region of *left* panel). The location of Mushroom body is masked with *yellow* dotted lines; Eiger, *red*; recruited plasmatocytes, *green*; transgenic brain, *blue*. **e**–**g** qRT-PCR analysis of eiger expression **e** and immunostaining **f** as well as western blot analysis **g** of JNK phosphorylation in brains of the transgenic flies with or without hemocyte-deficient background. Quantitative data are presented as the mean±SD of three independent experiments. *elav*>*Aβ42* transgenic or control (*elav* alone) flies with or without *Ecc15* intestinal infection were analyzed at 10 dpi for plasmatocyte recruitment, *eiger* expression, and JNK activation. *P < 0.05, ***P < 0.001; NS, not significant, H&E, haematoxylin and eosin. Scale bars, 50 μm

We next sought to identify the signaling relay that transduced this intestinal microbial infection signal to the transgenic brain. We suspected that peripheral immune cells might mediate this inter-organ communication based on the fact that one of our previous investigations showed that immune hemocytes conveyed enteric dysbiosis signals to a distant organ, eliciting innate immune responses in *Drosophila* ^15^ and that recruitment of immune hemocytes to the brain has been implicated in the progression of AD^23^. We first examined whether immune hemocytes were recruited to the brains upon *Ecc15* enteric infection by labeling plasmatocytes, functional macrophages in *Drosophila*, with an anti-NimC1 antibody^24^. Remarkably, a number of plasmatocytes were found to be recruited to the postero-dorsal site of the brain near the mushroom body lobe, a brain region involved in learning and memory (Fig. 3a). Similar results were observed when analyzing *NimC1* transcripts (Supplementary Fig. 8) or infection with a more pathogenic enterobacteria (*P. entomophila*; Supplementary Fig. 9), suggesting that enteric infection induces hemocyte recruitment to the transgenic brain. Therefore, we suspected that the recruited hemocytes might infiltrate into the brain to target amyloid-β plaques, as has been suggested previously^25–27^. However, after analyzing the z-stack confocal images of NimC1 immunostained brains, we found no sign of brain infiltration. In fact, the recruited plasmatocytes only localized in close proximity to the neural lamella (Supplementary Fig. 10), suggesting that the recruited hemocytes did not directly affect neurodegeneration from inside the brain.

To investigate whether the recruited hemocytes contributed to the progression of AD, we genetically depleted hemocytes in the transgenic fly after enteric infection. A hemocyte deficient *Drosophila* mutant (*dom* ^1^/*l(3)hem* ^*2*^) showed markedly diminished neurodegeneration and improved survival even when exposed to enteric infection (Fig. 3b,c), suggesting that the recruited hemocytes were definitely involved in neurodegeneration. We therefore reasoned then that the recruited immune hemocytes triggered the TNF-JNK pro-apoptotic signaling pathway in the brain, since JNK was readily activated in the transgenic brain by enteric infection (Fig. 2a–c). To examine whether the recruited hemocytes were associated with Eiger/TNF expression, we performed a double immunofluorescence staining of Eiger and NimC1. Indeed, much of the TNF signaling occurred in conjunction with, but not limited to, the recruited plasmatocytes (Fig. 3d). Importantly, the hemocyte deficient *dom* ^*1*^/*l(3)hem* ^*2*^ mutant showed decreased *eiger* expression and JNK activity in the brains of transgenic flies exposed to enteric infection (Fig. 3e–g), indicating that enteric infection promoted brain recruitment of hemocytes, triggering TNF-JNK signaling and the exacerbation of AD.

### Hemocyte recruitment involves a gut-brain cooperation

**Fig. 4:**
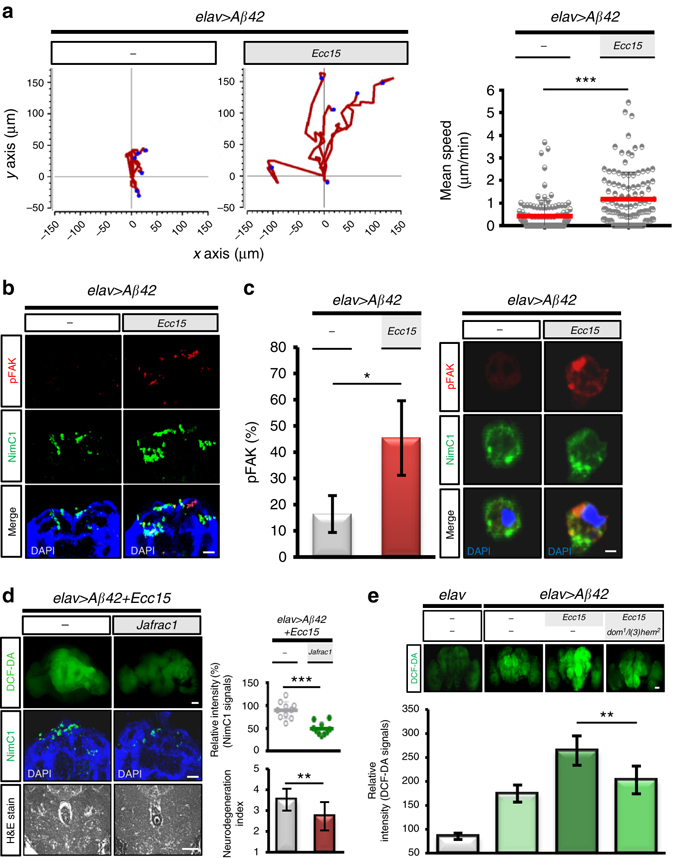
Gut–brain axis mediates the mobilization of hemocytes and their attraction to the transgenic brain in promoting neurodegeneration. **a** Time-lapse analysis of plasmatocyte migration after enteric infection. Representative examples of the migration trajectories of plasmatocytes (*n* = 6 in each group). **b**, **c** Coimmunostaining of phospho-FAK and NimC1 in plasmatocytes recruited transgenic brains **b** or in circulation **c**. Phospho-FAK, *red*; plasmatocytes, *green* (*n* = 15 in each group). **d** DCF-DA (*upper* panel) and NimC1 (*middle* panel) staining and histology (*bottom* panel) in the transgenic brains with or without overexpressing Jafrac1. **e** DCF-DA staining in transgenic brains with or without hemocyte deficient background. *elav* > *Aβ42* transgenic or control (*elav* alone) flies with or without *Ecc15* intestinal infection were analyzed at 10 dpi. DCF-DA signal, ROS stress; NimC1 signal, plasmatocyte. Brain histology, *n* = 10 each group. Quantitative data are presented as the mean±SD of three independent experiments. *P < 0.05, **P < 0.01, ***P < 0.001; NS, not significant, H&E, haematoxylin and eosin. Scale bars, 50 μm **b**, **d** and **e**; 5 μm **c**

Lastly, we became interested in how enteric infection induces hemocyte recruitment to the brains. We initially suspected that this might be caused by an increase in total plasmatocyte population. However, quantitative analysis of the total plasmatocyte population in the whole-body showed no noticeable difference in population size compared with non-infected controls (Supplementary Fig. 11). We then hypothesized that cell mobility of plasmatocytes might be increased facilitating migratory behavior. A time-lapse in vitro cell motility assay revealed that the plasmatocyte motility was significantly increased by the enteric infection (Fig. 4a). In addition, focal adhesion kinase activity, which is required for immune cell migration, was also increased in the plasmatocytes recruited to the brain or in circulation (Fig. 4b, c). These findings suggest that enteric infection may create greater opportunity for the recruitment of plasmatocytes to transgenic brains by increasing their migratory behavior.

We speculated that the brain could also actively deliver signaling cues to attract the highly migratory plasmatocytes. Prompted by the observation that the ROS levels were markedly increased in transgenic brains upon enteric infection (Fig. 2h, i), we investigated whether this contributed to plasmatocyte recruitment, because ROS generated at wound/damage sites are known to attract immune cells^28, 29^. Overexpressing Jafrac1, a homolog of mammalian Prx1, in the transgenic brain to attenuate the ROS response to enteric infection, we found both plasmatocyte recruitment and neurodegeneration to be diminished (Fig. 4d). In contrast, when ROS level was increased by RNAi gene knockdown of Catalase in transgenic or wild type brain, plasmatocyte recruitment was increased (Supplementary Fig. 12a, b), suggesting that brain ROS plays a key role in attracting immune hemocytes toward transgenic brains. We also found that the recruited hemocytes conversely exacerbated ROS production by the transgenic brain as the hemocyte deficient mutant showed a marked decrease in ROS generation by the brain (Fig. 4e). Considered together, our findings provide compelling evidence of a functional link between the gut–brain axis mediating the mobilization of hemocytes and their attraction to the transgenic brain, modulating neurodegeneration.

## Discussion

Understanding how inter-organ communication is established in maintaining systematic homeostasis in the body and by what means the communication may contribute to human diseases is key to overall health and well-being. The current study conceptually advances our understanding of how the gut–brain crosstalk may involve in the modulation of inflammatory response and neurodegenerative progression in AD brain. Given the conservation of inter-organ communication pathways, it is enticing to speculate the possibility that enteric dysbiosis may contribute to human AD progression.

The main findings of this work also highlighted the importance of immune hemocytes as a signaling relay between the gut and brain in controlling AD progression. As noted previously, the recruitment of peripheral immune cells to the brain may be induced by the presence of inflammation/injury originating from the brain, such as ischemic stroke^30^, traumatic brain injury (TBI)^31^ and neurodegenerative diseases^23^. Accordingly, in a broad sense, the enteric infection-induced hemocyte recruitment to the AD brain is functionally equivalent to the stroke/TBI-induced focal recruitment of peripheral immune cells in eliciting brain inflammatory response. It is important to note that an elevated ROS level in the brain is known to be required for the recruitment of immune hemocytes^32^. Our current study supports this concept through genetic manipulation of the brain ROS level to diminish the detrimental effect of enteric infection on AD progression. In addition to intracellular ROS stress, brain secretion of a chemokine CCL2, has also been reported to be required for the recruitment of immune cells^33^. Intriguingly, the chemokine homolog and it’s receptor in *Drosophila* have not yet been identified^34^. Thus, it is of interest in the future to determine what functional counterparts of chemokine/chemoattractant in *Drosophila* may be released from the brain to stimulate immune cell homing in response to ROS stress.

Previous studies on Parkinson’s disease^35^, ischemic stroke^36^, and epilepsy^37^ report similar observations that immune cell recruitment to the brain contributed to the aggravation of proinflammatory response in the brain. In apparent contrast to this concept, a previous study reported that the recruitment of macrophages to the AD brain actually functions to help clear the build-up of amyloid β plaques, and limits the progression of AD pathology^25^. These confounding effects of immune cell recruitment on the neurodegenerative brain might make it difficult to interpret a simple model of action on disease progression. One might deduce from this fact that the effects of recruited immune cells on the degenerative brain is, initially, to help clean the deposition of misfolded proteins and limit the progression of neurodegeneration. However, when excessively or chronically activated, they can overproduce proinflammatory cytokines and oxidants to damage neuronal cells.

Because of its role in maintaining body homeostasis, the gut-brain interplay can exert both detrimental and beneficial effects on brain neuronal survival. Although this work only demonstrates a detrimental role of the gut-brain axis on AD, future studies may want to explore how the gut–brain axis might be used in a protective role in slowing progression of AD. Interesting, a recent report on germ-free APP transgenic mouse model supports this premise by noting that a reduction of amyloid pathology is associated with a sterile environment effect^38^. The findings of such studies may lead to the discovery of new therapeutic strategies for the treatment of AD by targeting cellular factors in the gut.

## Methods

### Fly strains and genetic crosses

The following fly strains were obtained from the Bloomington *Drosophila* Stock Center: *elav* ^*c155*^ *-Gal4* (BL458), *UAS-Aβ42* (BL33769), and *l(3)hem* ^2^/TM6B (BL6185). *UAS-eiger-RNAi* (VDRC108814), *UAS-eiger-RNAi* ^45252^ (VDRC 45252), and *UAS-Catalase-RNAi* (VDRC103591) were obtained from the Vienna *Drosophila* RNAi Center. *UAS-Jafrac1*, *dom* ^*1*^/Cyo was provided by Dr Jean-Luc Imler^39^, *Dpt-GFP* and *Drs-GFP* reporters from Dr Bruno Lemaitre^40^, and the *gstD-GFP* reporter^41^ from Dr Chun-Hong Chen. All fly stocks were raised on standard Bloomington medium at 25 °C.

The transgenic flies ectopically expressing human *Aβ42* in neurons driven by *elav* ^*c155*^ *-Gal4* was used as an alternative model of AD^17^ and flies carrying the *elav* ^*c155*^ *-Gal4* driver alone served as control. To monitor ROS response in transgenic or control flies, *elav* or *elav;Aβ42/Cyo* female flies were crossed with *gstD-GFP* (II) homozygous male flies to obtain *elav/Y;Aβ42/gstD-GFP* and *elav/Y;gstD-GFP/+* lines, respectively. To determine AMP responses in transgenic flies, *elav/Y;Aβ42/+*; *Drs-GFP/+* and *elav/Y;Aβ42/+*;*Dpt-GFP/+* were generated. *elav/Y*;*+/+*;*Dpt-GFP/+* and *elav/Y;+/+;Drs-GFP/+* were used as control flies for the experiments. To generate hemocyte-deficient amyloid transgenic flies for studying brain responses to enteric infection, *elav;Aβ42/Cyo* female flies were crossed with *dom* ^*1*^ */Cyo;l(3)hem* ^*2*^ */TM6B* male flies to obtain flies carrying *elav/Y;Aβ42/dom* ^1^ *;l(3)hem* ^2^ */+*. To decrease ROS stress in the transgenic flies, *elav;Aβ42/Cyo* female flies were crossed with *UAS-Jafrac1* to obtain *elav/Y;Aβ42/+;Jafrac1/+* flies. By contrast, to increase the ROS level in transgenic flies, *elav;Aβ42/Cyo* female flies were crossed with *UAS-Catalase-RNAi* male flies to obtain the *elav/Y;Aβ42/Catalase-RNAi* fly line. To knockdown the Eiger level in the transgenic fly brain, *elav/Y;Aβ42/eiger-RNAi* was generated.

### Intestinal infection

Newly eclosed flies were reared at 25 °C for 3 days and then aged at 29 °C for indicated days before being subjected to intestinal infection with enterobacteria. For oral infection with enterobacteria, *elav* > *Aβ42* transgenic or control (*elav* alone) flies were fed with regular fly food mixed with or without *Ecc15* at OD600 = 100 or *P. entomophila* at OD600 = 20, by which the weight/volume ratio was 5 g food/1 ml of a bacteria solution. The bacteria-containing fly foods were prepared fresh and replaced every 2 days. The infected flies were kept at 29 °C for optimum growth of bacteria till assays.

### Immunostaining of plasmatocytes

The plasmatocytes that were recruited to the brains or in circulation were immunostained with anti-NimC1 and anti-pFAK antibodies. Circulating hemocytes were collected from the transgenic flies by cutting heads out of the bodies to exude the hemolymph. The collected hemocytes were plated on 0.01% poly-L-lysine-coated coverslips (12 × 12 mm) for 40 min, fixed (4% paraformaldehyde) for 20 min and permeabilized (PBS buffer with 0.2% Triton x-100) for 10 min, followed by blocking in bovine serum (PBS buffer with 0.2% Triton x-100 and 2% BSA) for 30 min at room temperature (RT). The hemocytes were then immunostained with anti-NimC1 or anti-pFAK antibodies at 4 °C overnight. After washing, the cells were incubated with FITC or Cy5-conjugated secondary antibodies (Jackson ImmunoResearch, 1:200) at 4 °C overnight and then counterstained with DAPI (1:500) at RT for 30 min prior to mounting for confocal microscopy. The dissected brains were fixed with 4% paraformaldehyde for 50 min at RT. After washing with PBS buffer, brains were permeabilized (PBS buffer containing 0.1% sodium citrate and 0.1 % Triton-x-100) for 30 min at RT and subsequently incubated with blocking buffer (washing buffer containing 2% BSA) for 1 h at 4 °C prior to performing immunostaining for anti-NimC1 and anti-pFAK. Quantification of the fluorescence intensities of NimC1 were determined using ImageJ.

### Lifespan and locomotion assay

Amyloid transgenic and control male flies at an age of 3 days were continuously infected with enterobacteria. Each vial housed 30–35 flies reared at 29 °C on a diet mixed with or without enterobacteria *Ecc15* (OD600 = 100) or *P. entomophila* (OD600 = 20). The dead flies were counted daily in each vial until all flies were dead. The assays were repeated in triplicate. Behavioral analysis of locomotor activity in transgenic, or control flies was assayed using 13-day-old male flies (total 120 flies/each group) with or without enterobacteria infection. The negative geotaxis response was assayed in plastic rearing vials (2.5 cm in diameter and 9.5 cm in height) by video recording the vertical climbing distance (<2 cm, 2 – 4 cm, or  > 4 cm) for 30 sec at RT when flies were tapped to the bottom of vial.

### ROS stress measurement

The brains of transgenic or control flies were freshly dissected out in PBS buffer for analysis of the intracellular ROS level by incubating with 10 μM DCF-DA fluorescent dye (Sigma) for 5 min at RT. The fluorescent signals were observed using a Leica TCS SP5 II confocal microscope with 20× objective at 490 nm excitation and 525 nm emission wavelengths. The whole-brain fluorescent signals were determined using ImageJ for quantification of the ROS level. Representative images were processed using Adobe Photoshop. To determine the brain ROS activities, *gstD-GFP* reporter expressions in transgenic or control flies were analyzed by western blotting of total brain lysates using an anti-GFP antibody. To determine whether the *gstD-GFP* fluorescent reporter expression was localized to neurons, double immunofluorescence staining of brains with antibodies against GFP and ELAV was performed and observed by confocal microscope with 63× oil objective.

### Cell migration analysis

Circulating hemocytes were collected from transgenic or control flies with or without *Ecc15* intestinal infection for 10 days at 29 °C. The hemocytes were placed on 0.2% gelatin-coated coverslips (12 × 12 mm), which were positioned in a 24-well culture plate, to adhere for 1 h at RT. Real-time imaging was performed using a time-lapse microscopic system (Leica AF 6000 LX wide-field fluorescence microscope). The time-lapse images were taken every 10 min for 3 h and then processed by ImageJ software. Cell migration were tracked manually with an ImageJ plugin (http://rsb.info.nih.gov/ij/plugins/track/track.html) for the measurement of basic track statistics. Tracking results were then processed with Chemotaxis and Migration tool V2.0 (http://ibidi.com/software/chemotaxis_and_migration_tool/) to create the xy coordinate plots and distance measurements.

### Brain histology

About 3 to 5 heads were used for each histological experiment. The heads of *elav* > *Aβ42* transgenic or control (*elav* > *Aβ42*) flies with or without continuous infection of enterobacteria for 14 days were dissected out for pre-embedding with 1% agar and then placed in 10% buffered formalin overnight, followed by standard paraffin-embedding histological procedures. Standard H&E staining was carried out on 5 μm-thick sections. Imaging of the sections was conducted with the use of a light microscope under white light. All experiments were performed in triplicate.

### Apoptosis assay

A total of 10–15 adult brains of transgenic or control flies with or without *Ecc15* intestinal infection were dissected out and stained with 1.6 × 10^−6^ M acridine orange dye (Sigma-Aldrich) at RT for 5 min. After washing with PBS, the brains were stained with Hoechst dye (1:500) at RT for 30 min. Additionally, the dissected brains of *elav*, *elav* > *Aβ42*, or *elav* > *Aβ42, eiger-RNAi* flies were also used to perform immunostaining for active caspase 3 and TUNEL (Clontech, ApoAlert DNA Fragmentation Assay Kit) following the manufacturer’s instructions. Quantification was performed by counting the number of active caspase 3-positive cells per brain.

### Western blotting

Adult heads of indicated genotypes were dissected out for collecting whole-cell lysates, which were separated using 16.5% Tricine-SDS-PAGE (for detection of Aβ42) or 12% SDS-PAGE (for detection of JNK, JNK phosphorylation or GFP proteins). The gel was then transferred to a PVDF membrane, probed with primary antibody overnight at 4 °C, and incubated with secondary antibody of anti-rabbit or anti-mouse IgG horseradish peroxidase (Jackson ImmunoResearch, 1:10,000) for 1 h at RT, followed by exposure to X-ray film. The full uncropped scans of western blots are shown in Supplementary Fig. 13.

### Primary antibodies

Primary antibodies and titers used in this study for immunostaining are as follows: Rabbit anti-Cleaved Caspase-3 (Asp175) antibody (Cell Signaling, 1:200), mouse anti-Elav antibody (Developmental Studies Hybridoma Bank, DSHB, 1:50), mouse anti-Repo (Developmental Studies Hybridoma Bank, DSHB, 1:50), rabbit anti-GFP (GeneTex, 1:200), mouse anti-NimC1 (a gift from Dr István Andó, 1:30)^24^, rabbit anti-Eiger (a gift from Dr Chun-Hong Chen, National Health Research institute, NHRI, 1:200), rabbit anti-FasII (a gift from Dr Vivian Budnik, 1:5000)^42^ and rabbit anti-pFAK (Cell Signaling, 1:200). For western blotting, mouse anti-Aβ42 (6E10) (Covance, 1:5000), rabbit anti-phospho JNK (pTPpY) (Promega, 1:2000), rabbit anti-JNK (Santa Cruz, 1:5000), and rabbit anti-GFP (GeneTex, 1:5000) were used.

### Quantitative RT-PCR analysis

SYBR green-based (Applied Biosystems) quantitative RT-PCR analysis was used for detection of AMP transcripts. The cDNAs were synthesized by reverse transcriptase with High Capacity cDNA RT kits (Applied Biosystems) using total RNA extracted from fly heads. The relative mRNA expressions of target genes were normalized to the housekeeping gene *rp49*. Fold induction was determined by normalization to the control sample. The following primers were used in this analysis: *Dpt* forward 5′-GTTCACCATTGCCGTCGCCTTAC-3′, *Dpt* reverse 5′-TTGTTCGCCCTCTTCGCTGTCCT-3′; *Drs* forward 5′-TTGTTCGCCCTCT TCGCTGTCCT-3′, *Drs* reverse 5′-GCATCCTTCGCACCAGCACTTCA-3′; *rp49* forward 5′-AGATCGTGA AGAAGCGCACCAAG-3′, *rp49* reverse 5′-CACCAGGA ACTTCTTGAATCC GG-3′; *CecA1* forward 5′-ATG AAC TTC TAC AAC ATC TT CGT-3′, *CecA1* reverse 5′-ATT GTG GCA TCC CGA G-3′; *AttA* forward 5′- GTG GTG GGT CAG GTT TTC GC-3′, *AttA* reverse 5′- TGT CCG TTG ATG TGG GAG TA-3′; *NimC1* forward 5′- GCGAT TTATC GAGTG GT-3′, *NimC1* reverse 5′-CGCC T AGAGG TAGAA CG-3′; *egr* forward 5′-CTGCC GAGAC CCTCA AGC-3′, *egr* reverse 5′-AGATC GTTAG TGCGA GAATG-3′.

### Colony-forming unit (CFU) assay

About 10 intestines or heads of flies with or without *Ecc15* intestinal infection at 10 days postinfection were dissected out and placed into 1.5 ml tubes for homogenization with TissueLyser II (Qiagen). Serial dilutions of these homogenates were spread onto 100 μg/ml rifampicin-containing solid LB medium plates and incubated overnight at 29 °C. Total 50 intestines or heads were used for five independent experiments.

### Intestinal integrity assay

Integrity of intestinal barrier function was assessed by measuring the post-feeding distribution of a nonabsorbable blue food dye (FD&C blue dye#1). Male flies at 10 days postinfection with *Ecc15* were transferred to fresh food containing blue dye (2.5% w/v) for 12 h at 25 °C. The gut integrity was determined by quantifying the blue dye coloration seen in the body cavity after feeding.

### Statistical analysis

Experimental data were analyzed using Graph Prism. Statistical analysis of a difference between groups was performed using an unpaired *t*-test. A *P*-value < 0.05 stands for significant, <0.01 stands for very significant and <0.001 stands for extreme significant. No statistical methods were used to predetermine sample size. Experiments were not randomized.

### Data Availability

All relevant data are available from the authors upon request.

## Electronic supplementary material

Supplementary Information
