# Supplementary material for: Intestinal microbial dysbiosis aggravates the progression of Alzheimer’s disease in Drosophila
Source: Nat Commun. 2017 Jun 20;8:24. doi: 10.1038/s41467-017-00040-6 (PMC5478647; doi:10.1038/s41467-017-00040-6)
Supplement: Supplementary file 1 — Supplementary Information [file 41467_2017_40_MOESM1_ESM.pdf]

File name: Supplementary Information
Description: Supplementary Figures

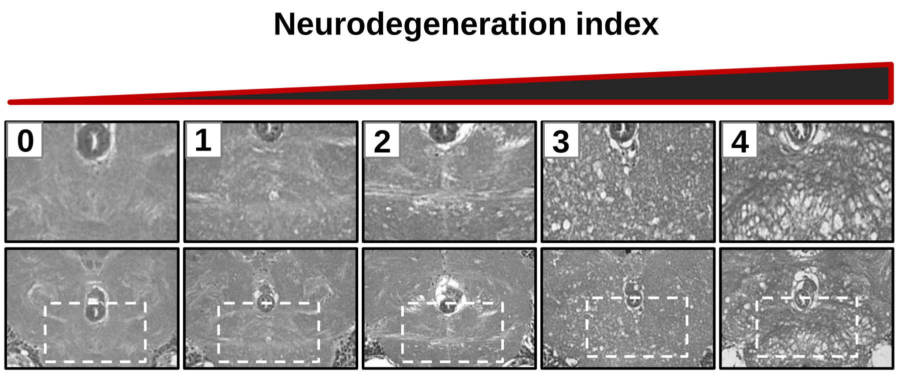

Supplementary Figure 1. The scoring system for quantification of neurodegeneration severity. The neurodegeneration index was defined as structural loss scored from 0 (no damage) to 5 (severe damage) based on the accumulated vacuolar lesions in the histological sections of neuropil of the central brain stained with haematoxylin and eosin (H&E).

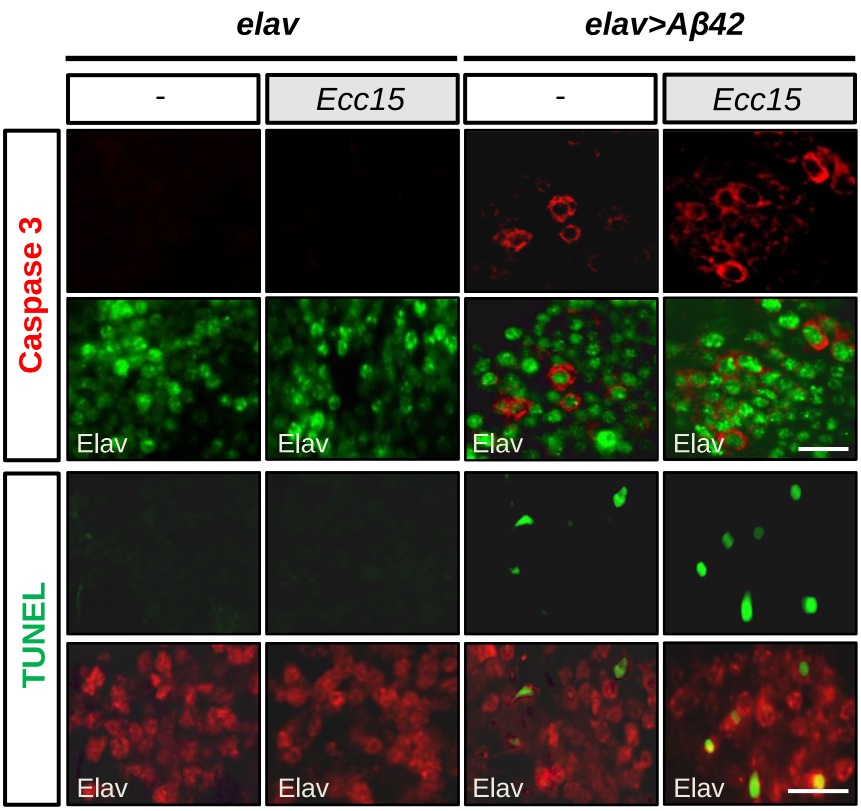

Supplementary Figure 2. Enteric infection aggravates neuronal apoptosis in amyloid transgenic fly brain. Confocal images showing neuronal apoptosis in *elav>Aβ42* transgenic fly brains. Transgenic flies (*elav>Aβ42*) or control (*elav* alone) with or without *Ecc15* intestinal infection were analyzed at 10 dpi. Active caspase 3, red; TUNEL, green. Figures are representative of three independent experiments. Scale bars, 10 μm.

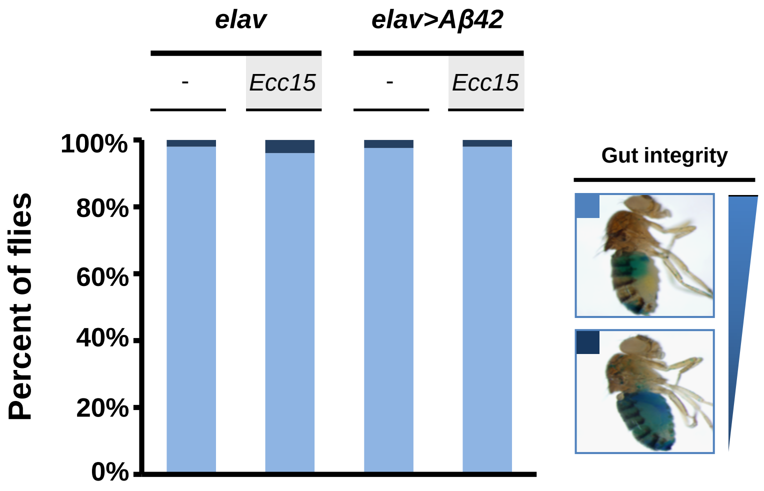

Supplementary Figure 3. *Ecc15* enteric infection causes no obvious intestinal barrier integrity loss. The intestinal barrier function was assessed by quantifying the distribution of the blue dye throughout the body cavity in *elav>Aβ42* transgenic or control (*elav* alone) flies with or without *Ecc15* enteric infection for 10 days. n=60 in each group.

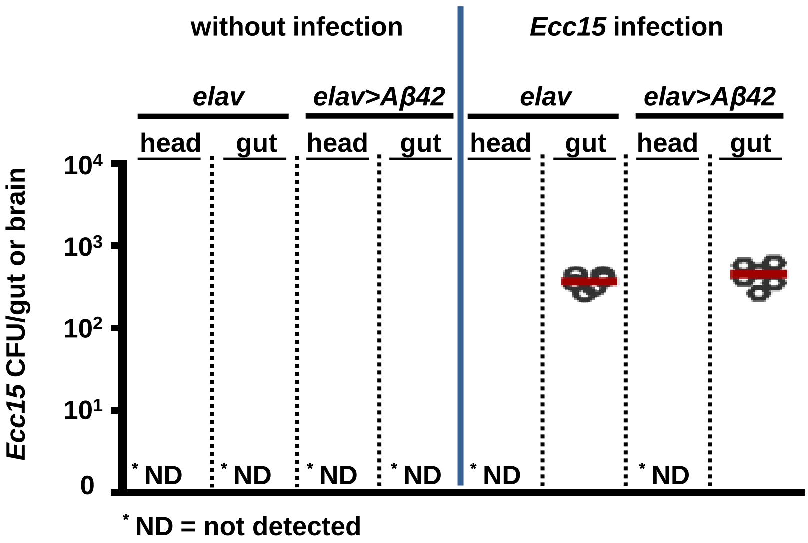

Supplementary Figure 4. Enterobacteria *Ecc15* is undetectable in amyloid transgenic fly brains after enteric infection. Colony formation unit (CFU) analysis of the whole gut or whole head from *elav>Aβ42* transgenic or control (*elav* alone) flies with or without *Ecc15* intestinal infection for 10 days. n=50 in each group. Quantitative data are presented as the mean±SD of five independent experiments.

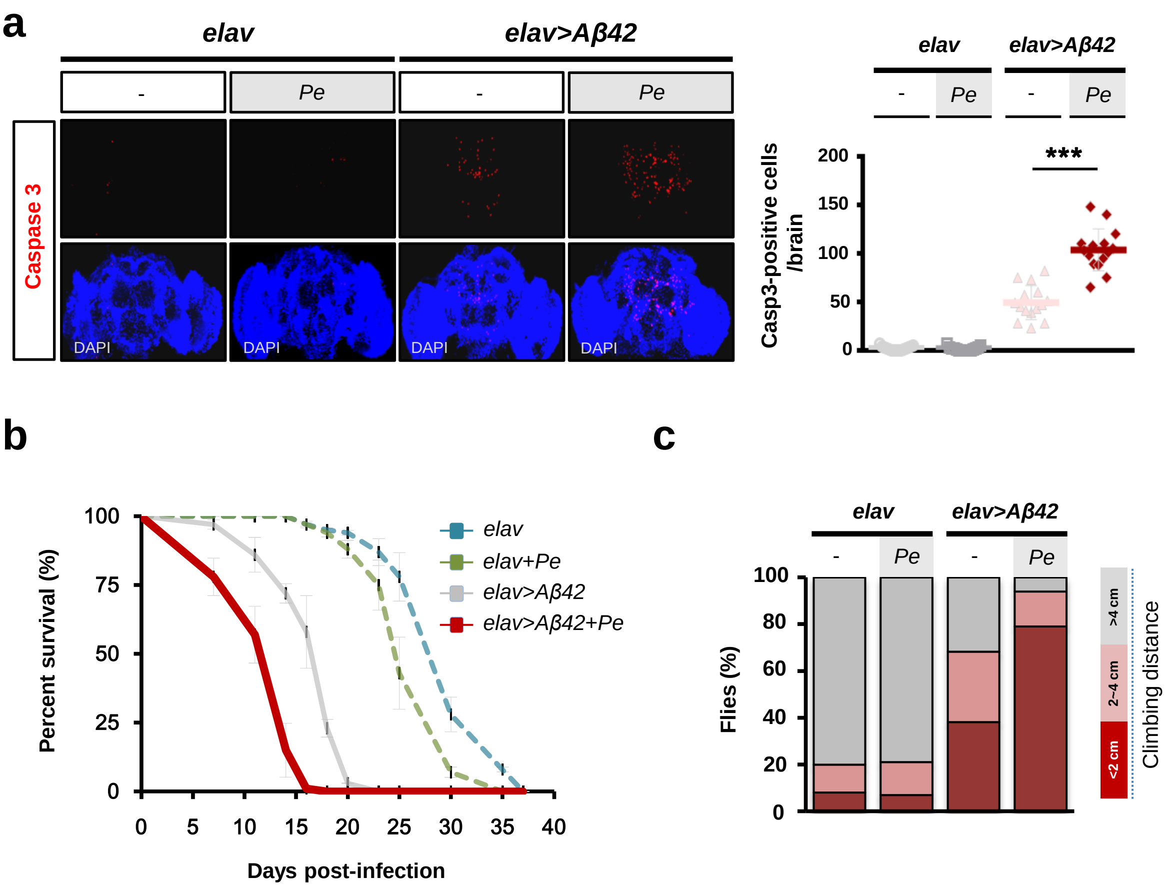

Supplementary Figure 5. *P. entomophila* (*Pe*) enteric infection also aggravates AD phenotypes in the fly. (a-c) Phenotypes of brain neuronal apoptosis (n=15 in each group) (**a**), lifespan (n=100 in each group) (**b**), and locomotion (n=120 in each group) (**c**) are aggravated by enteric infection with *P*. *entomophila* in *elav>Aβ42* transgenic flies. Assays were conducted at 10 dpi. Quantitative data are presented as the mean±SD of three independent experiments. ****P<0.001*. Scale bar, 50 μm.

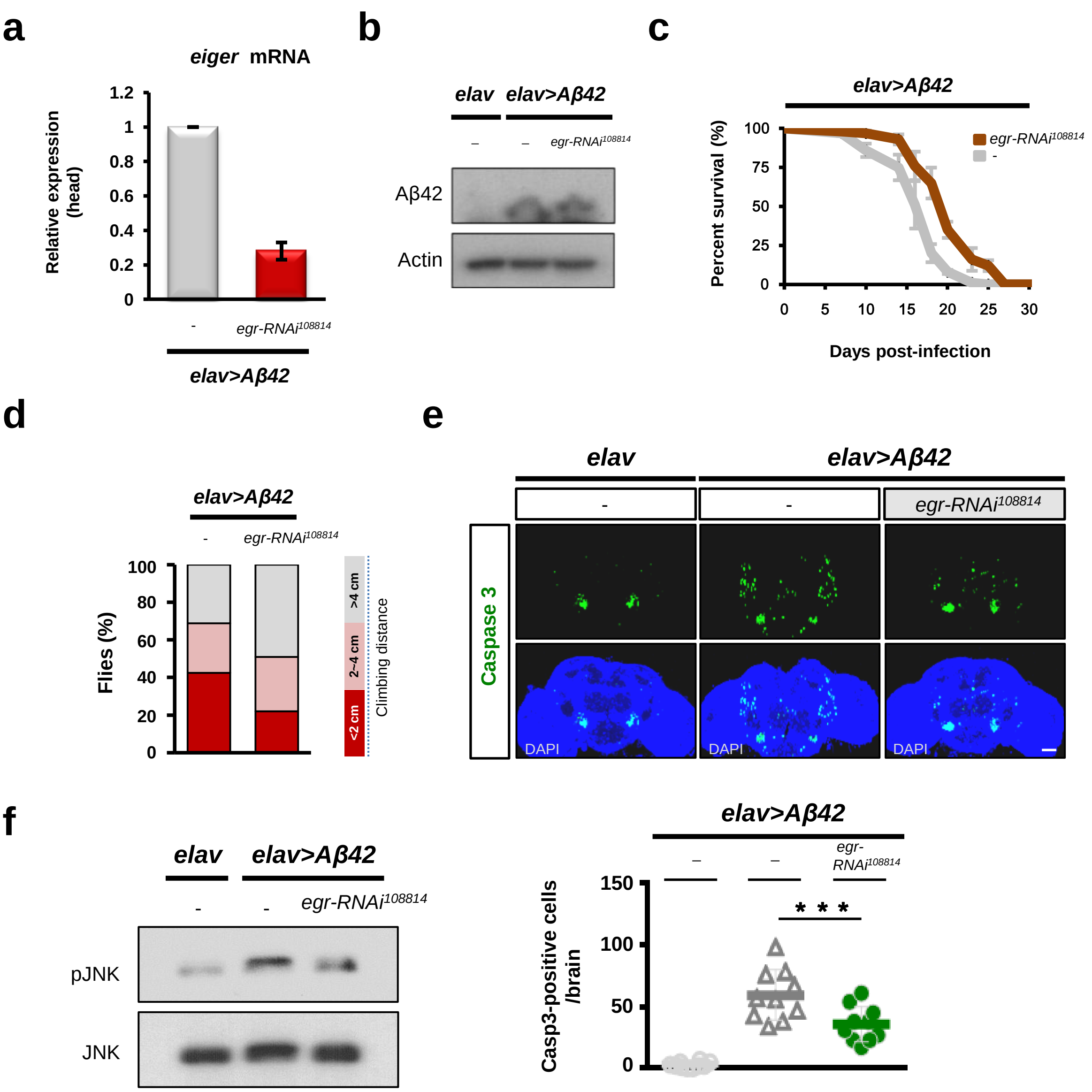

Supplementary Figure 6. Loss of Eiger/TNF rescues amyloid-induced neurodegeneration in adult flies without enteric infection. (**a**) *eiger* RNAi knockdown efficiency in the amyloid transgenic fly brain normalized with mock control. **(b)** Western blot analysis showing comparable Aβ42 levels in the amyloid transgenic fly brains with or without RNAi knockdown of *eiger*. (**c-f)** lifespan (**c**), locomotion behavior (**d**), caspase 3-dependent apoptosis (n=10 in each group) (**e**) and phosphorylation of JNK (**f**) in the brains of *elav>Aβ42* or *eiger*-depleted (*elav>Aβ42*, *egr-RNAi*) flies. Data presented as the mean±SD of three independent experiments. *** *P<0.001.* Scale bar, 50 μm.

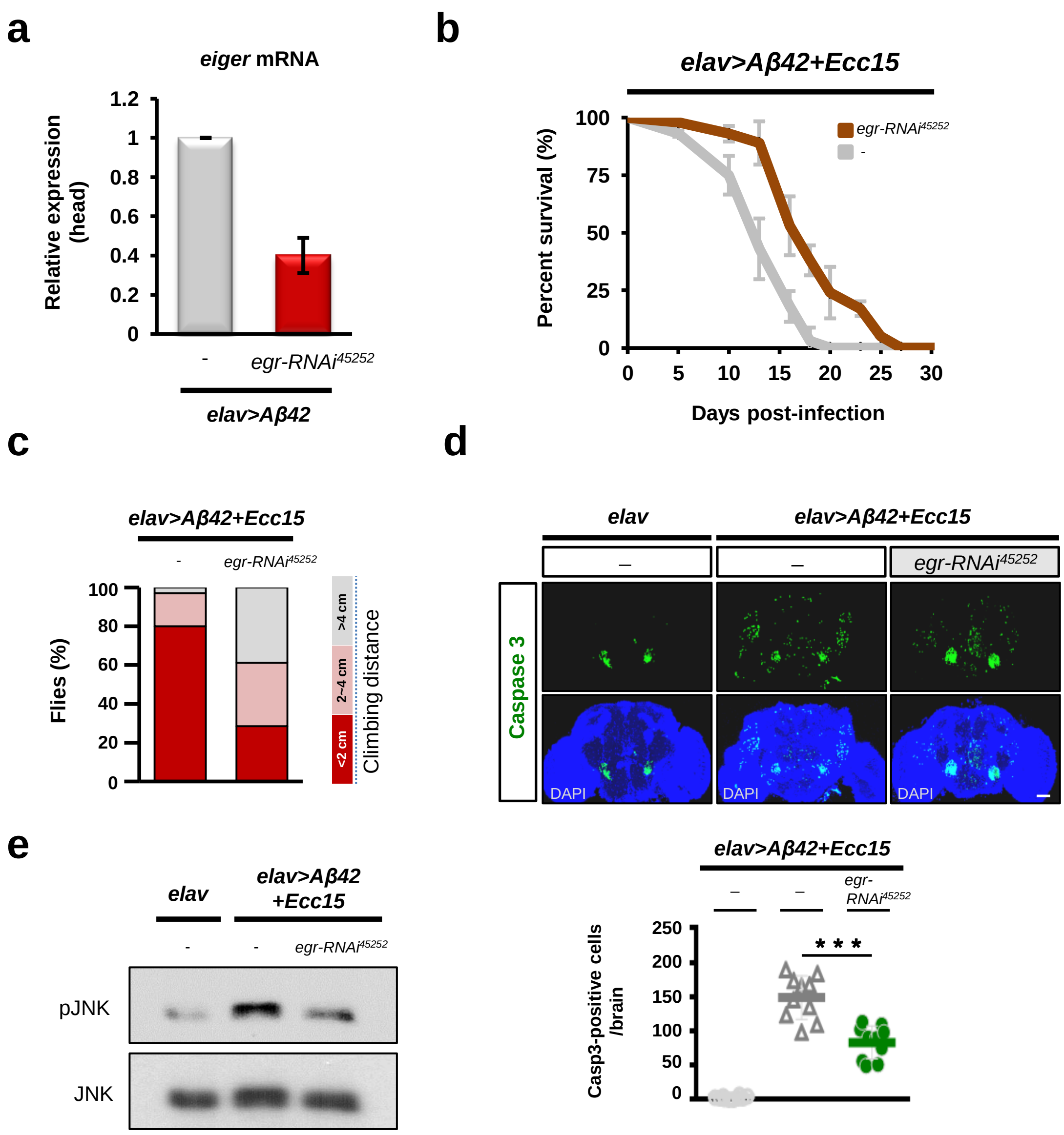

Supplementary Figure 7. A second eiger-RNAi fly line (VDRC45252) confirming the rescuing effect of RNAi-mediated knockdown of *eiger* for the enteric infection-exacerbated neurodegeneration in the Aβ42 transgenic fly. (**a**) qPCR analysis of knockdown efficiency mediated by elav>*eiger*-RNAi in the amyloid transgenic fly brain. **(b-e)** Lifespan (**b**), locomotion behavior (**c**), caspase 3-dependent apoptosis in brain (n=10 in each group) (**d**), JNK phosphorylation in brains (**e**) of *elav>Aβ42* or *eiger*-depleted (*elav>Aβ42*, *egr-RNAi^{45252}*) flies. Data presented as the mean±SD of three independent experiments. ****P<0.001.* Scale bar, 50 μm.

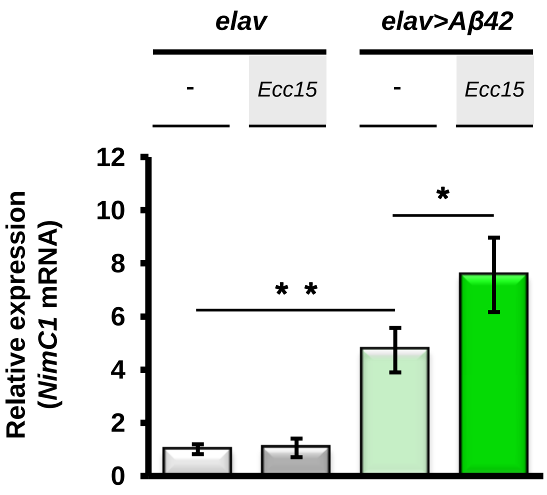

Supplementary Figure 8. Enteric infection encourages the recruitment of plasmatocytes to the amyloid transgenic brain. The *NimC1* transcript level was used as an indicator of plasmatocyte recruitment in the brain. *NimC1* transcript levels in brains of *elav>Aβ42* or control (*elav* alone) flies with or without *Ecc15* intestinal infection for 10 days were analyzed by qRT-PCR (n=60 in each group). Data presented as the mean±SD of three independent experiments. * *P<0.05,* ** *P<0.01.*

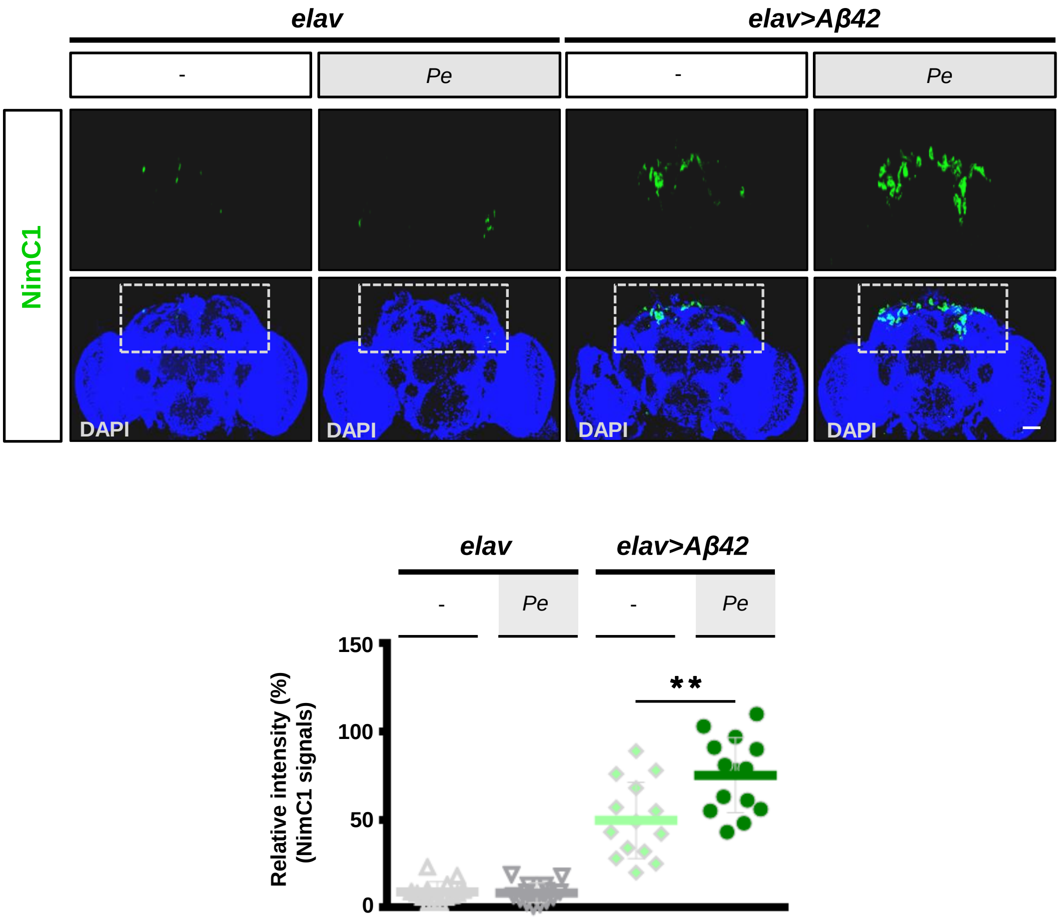

Supplementary Figure 9. Enteric infection of *P. entomophila* promotes plasmatocyte recruitment to the amyloid transgenic brain. Confocal immunohistochemistry analysis showing plasmatocyte recruitment to the brains (upper panel). Plasmatocytes (NimC1 signals), green; DAPI, blue. Quantitative data are presented as the mean±SD of three independent experiments (n=15 in each group; lower panel). *elav>Aβ42* flies or control (*elav* alone) with or without *P. entomophila* intestinal infection for 10 days. ** *P<0.01.* Scale bar, 50 μm.

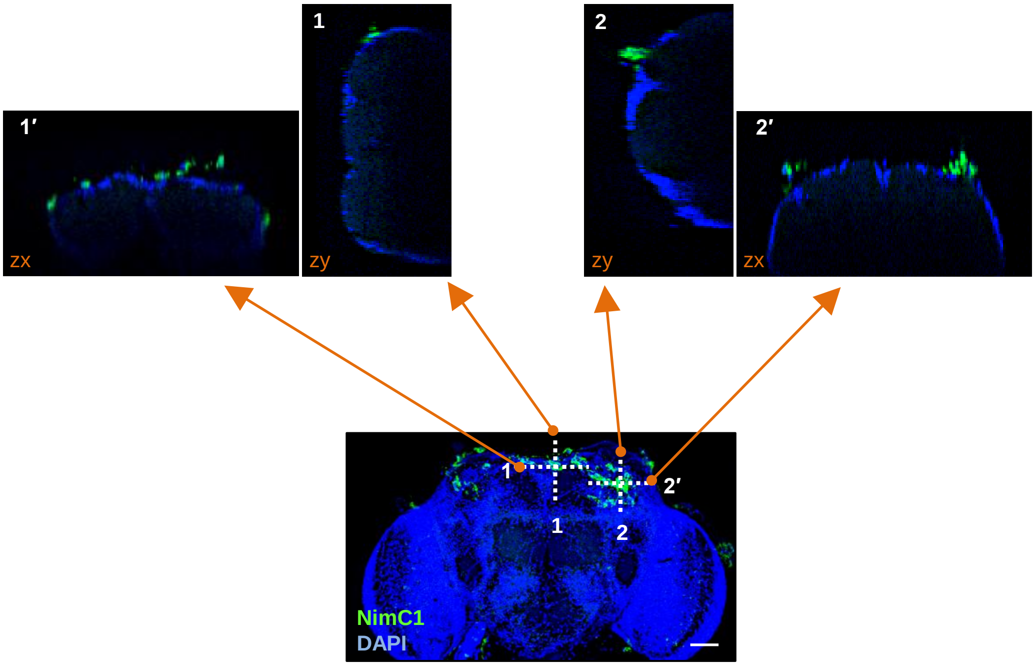

Supplementary Figure 10. No sign of brain infiltration with plasmatocytes was observed upon enteric infection. The z-stack confocal images of NimC1 signals in the amyloid transgenic fly brain were analyzed. Two regions (1&2) in the brain were sampled for zy and zx axis analysis. Plasmatocyte (NimC1 signal), green; brain morphology (DAPI), blue. Scale bar, 50 μm.

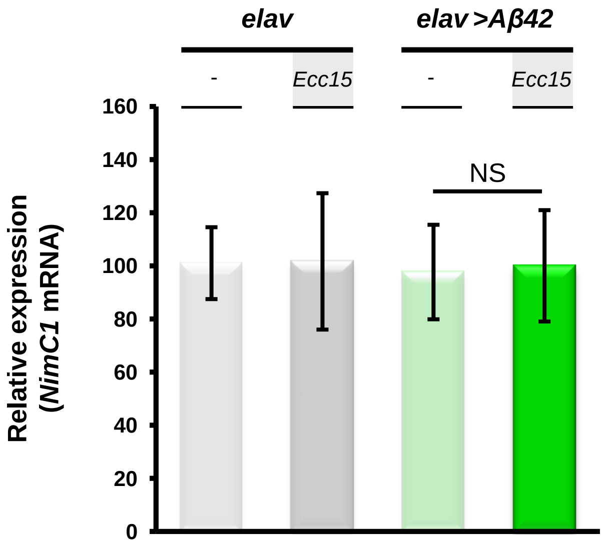

Supplementary Figure 11. The total population of plasmatocytes was not noticeably affected by the enteric infection with *Ecc15*. The total number of plasmatocytes was quantified by analyzing the *NimC1* transcript. qRT-PCR quantification of the *NimC1* transcript level in whole flies of the indicated genotypes treated with or without *Ecc15* enteric infection for 10 days (n=30 in each group). Data presented as the mean±SD of three independent experiments. *NS*, not significant.

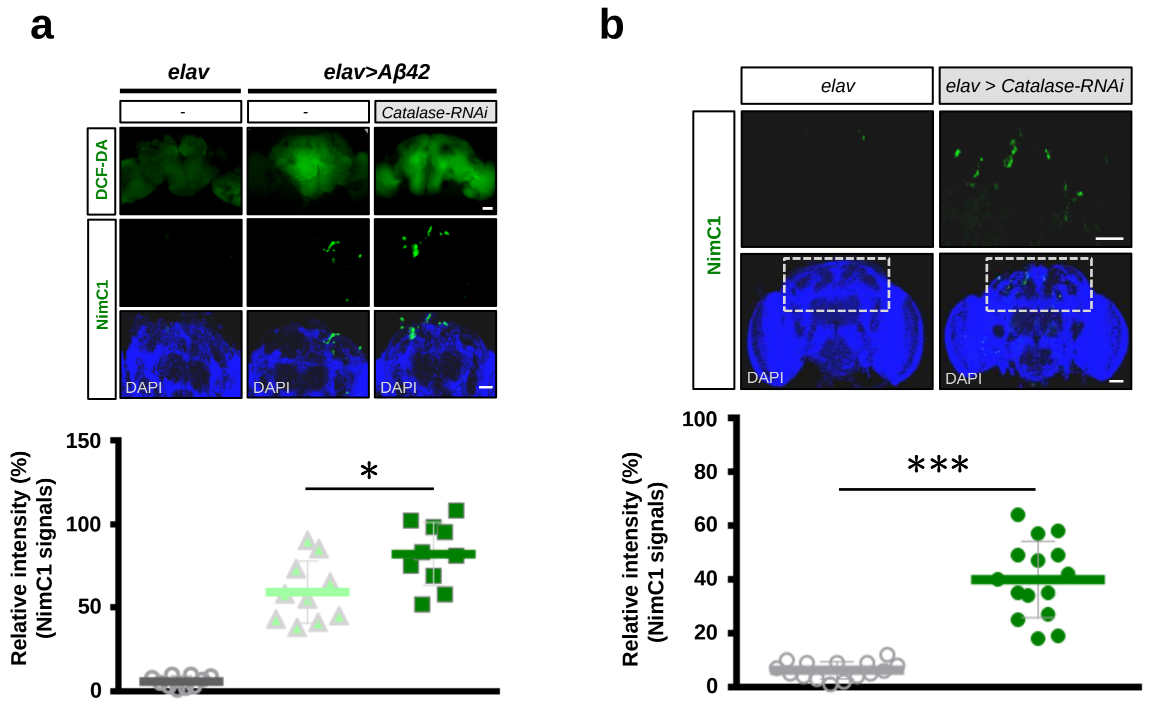

Supplementary Figure 12. Elevation of ROS level by RNAi gene knockdown of Catalase in the brain stimulates plasmatocyte recruitment to the brain. (a,b) Confocal images showing plasmatocytes (green, NimC1 signals) recruitment to the brains of *elav>Aβ42* flies (n=10 in each group) (**a**) or control flies (*elav* alone) (n=15 in each group) (**b**) with or without RNAi-mediated depletion of *Catalase* from the brain. Quantitative data are presented as the mean±SD (lower panels). * *P<0.05,* * * * *P<0.001.* Scale bars, 50 μm.

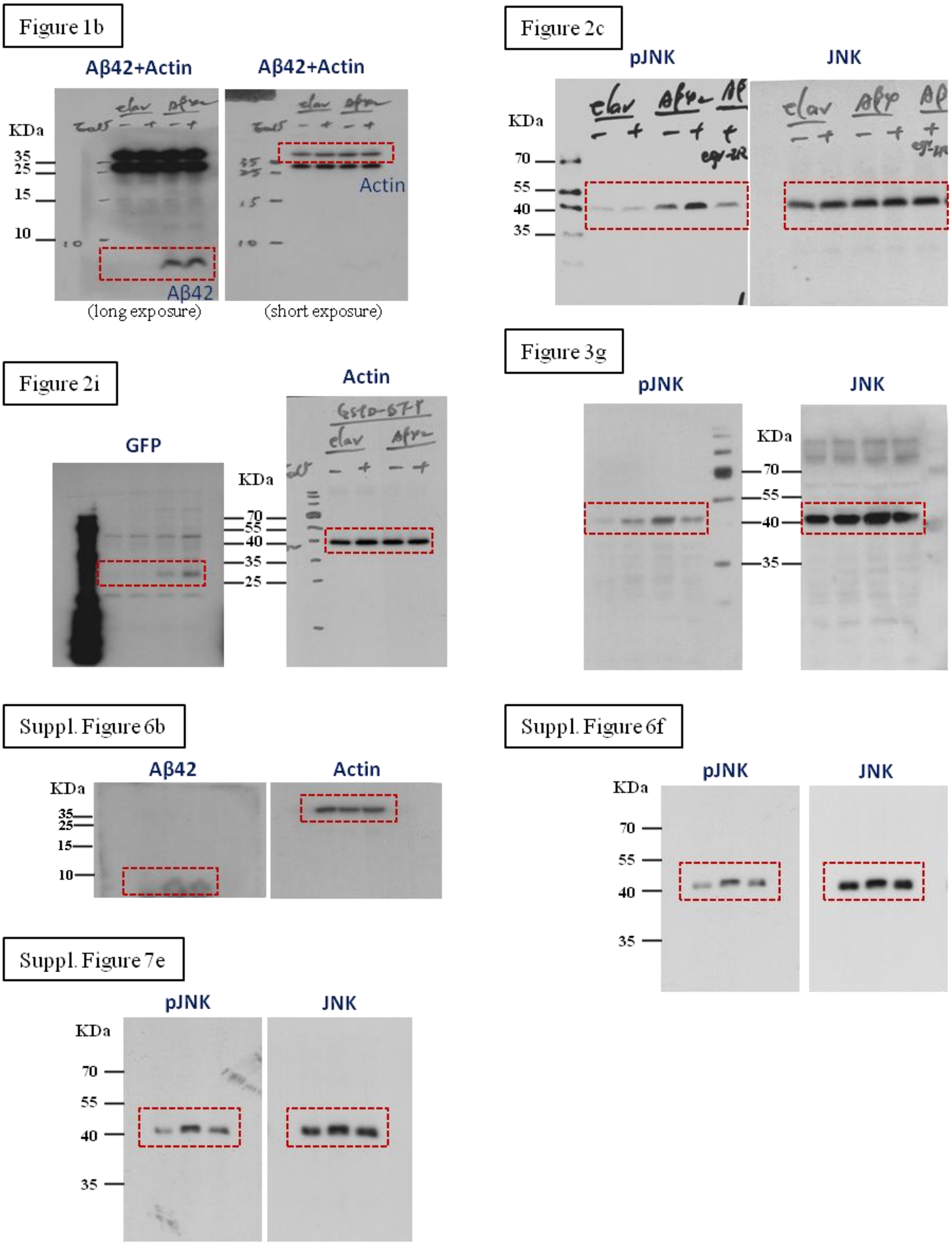

Supplementary Figure 13. Full uncropped scans of western blots for main figures 1, 2, and 3 and supplementary figures 6 and 7.
